# *Lacticaseibacillus rhamnosus* modulates the inflammatory response and the subsequent lung damage in a murine model of acute lung inflammation

**DOI:** 10.1016/j.clinsp.2022.100021

**Published:** 2022-03-15

**Authors:** Fabiana Olimpio, José Roberto Mateus da Silva, Rodolfo P. Vieira, Carlos R. Oliveira, Flavio Aimbire

**Affiliations:** a Department of Medicine, Programa de Pós-graduação em Medicina Translacional, Escola Paulista de Medicina, Universidade Federal de São Paulo (UNIFESP), São Paulo, SP, Brazil; b Institute of Science and Technology, Programa de Pós-graduação em Engenharia Biomédica, Universidade Federal de São Paulo (UNIFESP), São José dos Campos, SP, Brazil; c Department of Human Movement Sciences, Universidade Federal de São Paulo (UNIFESP), Santos, SP, Brazil; d Institute of Science and Technology, Universidade Federal de São Paulo (UNIFESP), São José dos Campos, SP, Brazil

**Keywords:** Acute respiratory distress syndrome, Sepsis, Probiotic, Protective effect, Mice

## Abstract

**Objective:**

The present study investigated the anti-inflammatory effect of the probiotic *Lacticaseibacillus rhamnosus* (*Lr*) on lung inflammation induced by Lipopolysaccharide (LPS) of *Escherichia coli* in C57BL/6 mice.

**Methods:**

C57BL/6 mice were divided into four groups: control, LPS, *Lr* (1 day) + LPS, and *Lr* (14 days) + LPS. Total and differential cells from Bronchoalveolar Lavage Fluid (BALF) were counted in a Neubauer 40X chamber, and pro-and anti-inflammatory cytokines (IL-1β, IL-6, CXCL-1, TNF-α, TGF-β, and IL-10) were measured by ELISA assay. The analysis of whole leukocytes in blood was performed using the automated system Sysmex 800i. Morphometry of pulmonary tissue evaluated alveolar hemorrhage, alveolar collapse, and inflammatory cells. Pulmonary vascular permeability was assessed by Evans blue dye extravasation, and bronchoconstriction was evaluated in a tissue bath station. The transcription factor NF-kB was evaluated by ELISA, and its gene expression and TLR-2, TLR-4, MMP-9, MMP-12, and TIMP by PCR.

**Results:**

The probiotic *Lr* had a protective effect against the inflammatory responses induced by LPS. *Lr* significantly reduced pro-inflammatory cells in the airways, lung parenchyma, and blood leukocytes. Furthermore, *Lr* reduced the production of pro-inflammatory cytokines and chemokines in BALF and the expression of TLRs, MMPs, and NF-kB in lung tissue and maintained the expression of TIMP in treated animals promoting a protective effect on lung tissue.

**Conclusions:**

The results of the study indicate that pre-treatment with the probiotic *Lr* may be a promising way to mitigate lung inflammation in endotoxemia.

## Highlights

• *Lacticaseibacillus rhamnosus* prevents lung neutrophilia.
• *Lacticaseibacillus rhamnosus* attenuates bronchial hyperreactivity.
• *Lacticaseibacillus rhamnosus* reduces pulmonary oedema.

## Introduction

The Acute Lung Injury (ALI) and its most severe form, Acute Respiratory Distress Syndrome (ARDS), is one of the major chronic health conditions in which disability and death rates are increasing worldwide; however, the development of new strategies to disease management remains underwhelming.^1^ Although the intrinsic factors that contribute to ALI development are still unclear, the septic syndrome is recognized as a risk factor for the disease.^2^

In ALI, chemokines, and cytokines, such as CXCL1 as well as cytokines TNF, IL-1β, and IL-6, are chemotactic factors that attract inflammatory cells to the injured lung, principally neutrophils and monocyte-derived macrophage, where the pulmonary destruction initiates, compromising the alveolar parenchyma.^3^ This is because an immune response imbalance is characterized by specific transcription factors.^4^ Therefore, the increased NF-κB expression is associated with the differentiation of the Th1 response of pro-inflammatory cytokines that produce cells. The alteration in the expression or function of NF-κB may be associated with the pathogenesis of ALI.^4^ Recent studies suggest that during the disease, imbalances occur in the pattern of Th1/regulatory T-cells (Treg) for immune responses with high levels of pro-inflammatory cytokines and decreased IL-10 cytokine.^5^ Corroborating studies indicate that Th1 cells, producers of pro-inflammatory cytokines, can recruit neutrophils to the lung that contribute to the disease aggravation. Among the IL-10 producing cells are Tregs, which are important to balance immune responses and maintain immunological tolerance to antigens because IL-10 suppresses the pro-inflammatory response-associated genes.^6^

The exacerbated activity of Metalloproteinases (MMP) from neutrophils in ALI patients is responsible for the destruction of the alveolar parenchyma. Neutrophils release proteinases into lung milieu, such as metalloproteases MMP-9 and MMP-12, resulting in changes in lung architecture where the immune system promotes the perpetuation of inflammation in ALI.^7^ The effects of matrix MMP can be inhibited by Tissue Inhibitors of Metalloproteinase (TIMP) secreted by several cells. During the ALI pathogenesis, the balance between the effects of MMP and its TIMP is dysregulated since that MMP released by neutrophils overlaps with TIMP activity with consequent pulmonary tissue destruction.^8^

Lipopolysaccharide (LPS) from gram-negative bacteria, such as *Escherichia coli*, can directly trigger pathogen-associated molecular patterns such as Toll-Like Receptors (TLRs), particularly TLR2 and TLR4, to initiate pattern recognition. The expression of TLR2 and TLR4 is elevated in monocytes, and they are associated with a number of sputum neutrophils, secretion of pro-inflammatory cytokines, and lung function impairment.^9^ Some authors have shown that antagonists of both TLR2 and TLR4 attenuate lung inflammation and airway remodeling in septic mice[9] as well as in ALI patients.^10^

Due to the high morbidity and the limitations of existing ALI treatments,^11^ innovative action is needed against airway inflammation as well as alveolar collapse to better control the disease. One effective treatment for ALI may be to attenuate immune response driven by pro-inflammatory mediators and simultaneously upregulate the secretion of anti-inflammatory proteins in lung milieu.^6^ Therefore, the ability of probiotics to modulate the immune response and their effects in preventing the development of various lung diseases, including ALI, has caught the attention of many researchers.^12^

Microbe-induced changes appear key to orchestrating the multiple pathways involving T-cells, natural killer, and alveolar macrophages associated with the protective effect of probiotics.^13^ Studies in both animal models and clinical trials have identified immunomodulatory effects of many non-pathogenic bacteria and provided evidence that intestinal microbes can activate a common mucosal immune response and, thus, influence sites distant to the intestine, including the respiratory tract.^14^ The relationship between gut microbiota and lung inflammation development has been investigated by some authors who have demonstrated the interaction between microorganisms and their metabolites with dendritic cells, lung macrophages, and airway epithelial cells.^15^ In fact, a gut-lung axis can modulate both the immune and inflammatory responses in the lung environment.^16^

Respiratory effects of probiotics in animal models have included attenuating allergic airway responses and protecting against respiratory pathogens.^17^ Some authors have evidenced that *Lactobacilli* modulate gut microbiota and reduce enteritis and ventilator-associated pneumonia in septic patients.^18^ In addition, the oral administration of *Lactobacillus* decreases lung inflammation and increases pulmonary functions in septic patients.^19^ Moreover, probiotic *Lactobacillus* administrated to children with ALI alleviates lung inflammation and improves pulmonary function.^20^ Nevertheless, few studies have focused on the effect of *Lactobacilli* on both molecular and cellular targets involved in ALI from the LPS-induced *in vivo* model of sepsis.

Some authors have shown that the action mechanism of beneficial probiotics involves the microbe-associated molecular patterns of the commensal bacteria recognized by TLRs present on the surface of recognition cells, whose activation through TLRs can induce Treg immunity and consequently the secretion of anti-inflammatory cytokines, such as IL-10.^21^ In this way, probiotics may induce Treg cells in gut lymphoid tissue that can spread to the airways in response to inflammation. Growing evidence from a range of model systems indicates the ability to induce Treg classes able to secrete IL-10, and that attenuated the Th1 responses may be a critical element in the anti-inflammatory effect of many probiotic organisms.^15^

Ultimately, considering that the effect of acute administration of *Lacticaseibacillus rhamnosus* on airway inflammation and lung damage in a murine model of ALI induced by lipopolysaccharide from *Eschericia coli* has not well been elucidated yet, the present study was designed to investigate if the oral feeding with probiotic *Lacticaseibacillus rhamnosus* modulates the equilibrium between the secretion of pro-and anti-inflammatory cytokines and attenuates the lung damage, by modulating TLR and NF-κB, in response to the ALI.

## Material and methods

### Animals

Male C57Bl/6 mice, weighing approximately 20 g, were provided by the Centre for the Development of Experimental Models (CEDEME) of the Federal University of São Paulo. They were used and kept under controlled conditions of humidity, light, and temperature in the Experimentation vivarium of ICT-UNIFESP in São José dos Campos, SP. The study was approved by the Research Ethics Committee of the Federal University of São Paulo under opinion number 6844251018.

### Induction of ALI with LPS

The mice were anesthetized with ketamine (100 mg/kg) and xylazine (10 mg/kg) intramuscular received an intranasal instillation of LPS (0.5 mg/kg) 24 h before euthanasia.^22^ Lipopolysaccharide (LPS) obtained from *Escherichia coli* O111: B4 was purchased from SIGMA-ALDRICH. During preparation, 10.5 µg of LPS was diluted in 35 µL of PBS with Ph 7.2 sterile by autoclaving at 120°C for 15 min and filtered through a 0.22 µm membrane to each mouse.

### Oral feeding with probiotic

**Fig. 1:**
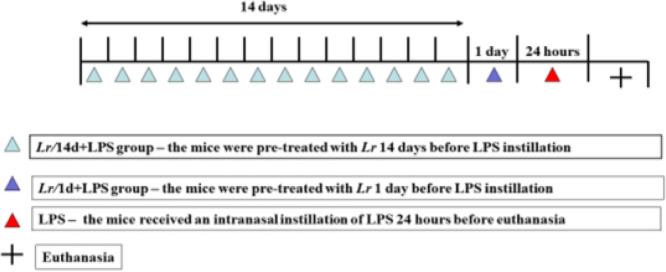
Times schedule of pre-treatment time with *Lacticaseibacillus rhamnosus* and induction of acute pulmonary inflammation. Male C57BL/6 mice were anesthetized with ketamine (100 mg/kg) and xylazine (10 mg/kg) intramuscular and exposed to *Escherichia coli* O111: B4 lipopolysaccharide (0.5 mg/Kg, intranasal, 24 h before euthanasia) to induce acute pulmonary inflammation. The mice were divided into two groups of *Lr* treatment (10-CFU/300µLPBS/mouse, orogastric by gavage), as described herein: *Lr*/1d+LPS group ‒ the mice were treated with *Lr* 1 day before LPS instillation; *Lr*/14d+LPS group ‒ the mice were treated with *Lr* 14 days before LPS instillation. The mice were euthanized 24h after the LPS.

Probiotic *Lacticaseibacillus rhamnosus* (*Lr*) lineage G-114 was purchased from the Vitalis handling pharmacy (CNPJ 67937706000155). During preparation, 1 × 10^9^ CFU (colony forming unit) of *Lr* were diluted in 300 µL of PBS (Phosphate-Buffered Saline) with Ph 7.2, sterilized by autoclaving at 120 °C for 15 min, and filtered in a 0.22 µm membrane for each mouse. The mice received 1 × 10^9^ CFU via gavage. The mice were randomly divided into two groups of *Lr* treatment, as described herein: *Lr/*1d+LPS group – the mice were treated with *Lr* 1 day before LPS instillation; *Lr/*14d+LPS group – the mice were treated with *Lr* 14 days before LPS instillation. The experimental protocol is illustrated in Fig. 1.

### Experimental groups

The mice were randomly divided into seven groups of 6 animals each: control (mice received PBS); *Lr/*1d (mice received *Lr* for one day); *Lr*/14d (received *Lr*for 14 days); LPS (mice received LPS); Lr/1d+LPS (mice received *Lr*1 day before LPS); Lr/14d+LPS (mice received *Lr* during 14 days before LPS).

### Total and differential count of cells infiltrated in the bronchoalveolar lavage fluid

The mice were anesthetized with ketamine (200 mg/kg) and xylazine (10 mg/kg) intraperitoneal and underwent the abdominal aorta bleeding procedure. During the collection of Bronchoalveolar Lavage Fluid (BALF), a tracheotomy was performed through a catheter (*n*= 18), and 0.5 mL of PBS was introduced, repeating the procedure three times. Then, the BALF was centrifuged at 3000 rpm for 15 min at 4 °C, and the supernatant was collected and stored in a freezer at 80 °C for the ELISA technique. The pellet was resuspended with 1 ml of sterile PBS and filtered for total cell count, 90 µL of a sample of centrifuged and resuspended BALF was used, and 10 µL of trypan blue in a 96-well plate. Cell counting was done in two random quadrants in a 40X Neubauer chamber. For the differential count, 150 µL of samples of centrifuged BALF were placed and resuspended in Cytospin (Excelsa® Flex 3400 ‒ Fanem) at 1500 RPM for 10 min.

### Leukocytes in blood

Five microliters of venous blood were collected by using vacuum tubes containing EDTA K2 as an anti-coagulant. The whole blood analysis (white and red series) was performed using the automated system Sysmex 800i (Sysmex Europe GmbH, Germany).

### Evaluation of inflammatory mediators in BALF and transcription factor NF-κB in lung tissue

The BALF (3 × 0.5 mL of PBS) was collected and centrifuged at 3000 rpm for 15 min at 4 °C, and then the supernatant was collected and resuspended in 1 mL of PBS. For the homogenate, 60 mg of tissue was weighed and macerated with 1 mL of PBS. Protein dosage was done by the Bradford method. The sample was stored in a freezer at -80 °C for analysis of cytokines (IL-1β, IL-6, TNF-α, IL-10, TGF-β, and CXCL-1) and NF-kB/NF-kBp65 by means of ELISA (Kit DuoSet®, R&D Systems) following the manufacturer's recommendations. For all Elisa techniques, the preparations of the reagents were performed according to the final volume of the samples following the manufacturer's recommendations.

### Morphometry: evaluation of inflammatory characteristics in the structure of pulmonary lobe tissue

After the induction of acute pulmonary injury, the animals were anesthetized and subsequently euthanized by the abdominal aorta bleeding procedure. Then their left lung lobe was removed and stored in 10% formaldehyde and sent to the laboratory specialized in histological analysis (Histocell, São Paulo, SP, Brazil). During the procedure, sagittal cuts were made over the pulmonary lobe along with other routine procedures for inclusion in paraffin. The lobes were then stained with Haematoxylin-Eosin (H&E). Analysis of histological sections was performed blindly to determine the pulmonary changes induced by acute pulmonary inflammation over the lung parenchyma, including alveolar collapse, interalveolar hemorrhage, inflammatory cell. The histological sections stained by H&E were analyzed in increments of 200 × in 15 fields and 400 × in 10 random fields of the microscope (OLYMPUS MODEL BX43F). After capturing the images, a colors filter was applied to all readings from all experimental groups in the IMAGE PRO PLUS program according to the program manual and required calibration. To calculate the parenchyma area (µm^2^), the air area was subtracted from the total image area.^23^ To calculate the percentage of hemorrhage,^24^ collapse^25^ multiplied the total area affected by 100 and divided by the tissue area. For the density of inflammatory cells, the number of cells (neutrophil, macrophage, and lymphocyte) was divided by the area of tissue, and the results were expressed as a number of cells/mm^2^.^26^

### Lung edema

Pulmonary vascular permeability was assessed by Evans blue dye extravasation. In brief, Evans blue dye (25 mg/kg) was given intravenously to mice 5 min before the animals were killed. 20 h after LPS inhalation, the mice were killed, the lungs were perfused as described above, and two samples of lung parenchyma were removed. Both were weighted, and then one was placed in formamide (4 mg/mL wet weight) at 20 C for 24 h, and the other was put to dry in an oven (60 °C) till constant weight. The concentration of Evans blue dye extracted in formamide was determined by spectrophotometry at a wavelength of 620 nm (SpectraMax i3, Molecular devices, CA, USA) using a standard dilution of Evans blue in formamide (0.3–100 mg/mL). The dry/wet ratio of each lung sample was determined (index of edema) and used in the final calculation of Evans blue extravasation, which was expressed as mg Evans blue/g of dry weight. The expression of the results as a function of the dry weight of tissue avoided under-evaluation of changes due to edema.^27^

### Bronchial hyperreactivity

After anesthesia, the bronchus was removed and cleaned from connective tissue. Each bronchus segment was suspended longitudinally between stainless steel triangular supports in 15 mL organ baths. The lower support was attached to the base of the organ, and the upper support was attached via a chain to a force transducer from which isometric tension was continuously displayed on a multichannel recorder (Biopac Systems Inc. Tissue Bath Station, CA, USA). Tissue samples were bathed in modified Krebs solution containing (mM) 125 NaCl, 14 NaHCO_3_, 4 KCl, 2.25 CaCl_2_, 2 H_2_O, 1.46 MgSO_4,_7 H_2_O, 1.2 NaH_2_PO_4_ H_2_O, and 11 glucose. The baths were ventilated with 5% CO_2_ in oxygen, a pH of 7.35–7.40 was maintained, and the temperature was held 37 °C. First, each tissue sample was passively stretched to a tension equal to 1.0 g weight to optimize the resting length of each Bronchus Smooth Muscle (BSM) segment. After this procedure, the passive resting tension of each BSM segment was set to a tension equal to 0.5 g weight. Measurement of BSM contraction forces was performed by varying the concentration of MCh-levels from 10^−10^to 10^−3^M.^28^

### Gene expression of TLR2 and TLR4 receptors; MMP-9 and MMP-12 metalloproteases; TIMP inhibitor; and NF-kB transcription factor by real-time quantitative PCR (qPCR)

Gene expression of TLR2 and TLR4 receptors; MMP-9 and MMP-12 metalloproteases; and NF-kB transcription factor was quantified by real-time reverse transcription-polymerase chain reaction using the Promega SV Total RNA Isolation System kit according to the manufacturer's instructions. Values were normalized by the expression of GAPDH and β-actin expressed by arbitrary units. The sequence of primers can be found in Table 1.

**Table 1.** Primer sequences used in qRT-PCR.

Primers	**Forward primer 5’-3’**	**Reverse primer 3’-5’**	**GenBank accession n°**
MMP-9	TGTACGGACCCGAAGC	CCGTCCTTATCGTAGTCAG	NM013599
MMP-12	TTTGACCCACTTCGCC	GTGACACGACGGAACAG	AK089523
TIMP-1	CCA CGA ATC AAC GAG ACC	GGC CCG TGA TGA GAA AC	NM011593
NF-kB	TCCGGGAGCCTCTAGTGAG	TCCATTTGTGACCAACTGAACGA	NM_008689.2
TLR-2	GAGCATCCGAATTGCATCACC	CCCAGAAGCATCACATGACAGAG	NM_011905.3
TLR-4	CATGGATCAGAAACTCAGCAAAGTC	CATGCCATGCCTTGTCTTCA	NM_021297.2
GAPDH	CACTCACGGCAAATTCAACGGCAC	GACTCCACGACATACTCAGCAC	AA661116.1
β-actin	TGCTGTCCCTGTAGTCCTCT	AGGTCTTTACGGATGTCAACG	NM 007393.1

MMP-9, Matrix Metalloproteinases-9; MMP-12, Matrix Metalloproteinases-12; TIMP-1, Tissue Inhibitor Matrix Metalloproteinase-1; NF-kB, Nuclear Factor kappa B; TLR-2, Toll-Like Receptor-2; TLR-4, Toll-Like Receptor-4; GAPDH, Glyceraldehyde-3 Phosphate Dehydrogenase; β-actin, Beta-Actin Gene.

### Statistical analysis

Statistical analyses were conducted using the GraphPad Prism v.6.0 software. For the treatment of the obtained data, the authors used the analysis of variance (ANOVA) followed by the Tukey post-test. The results indicated by "*p* < 0.05" were considered significant, with a 95% Confidence Interval.

## Results

### Cellularity in bronchoalveolar lavage fluid

**Fig. 2:**
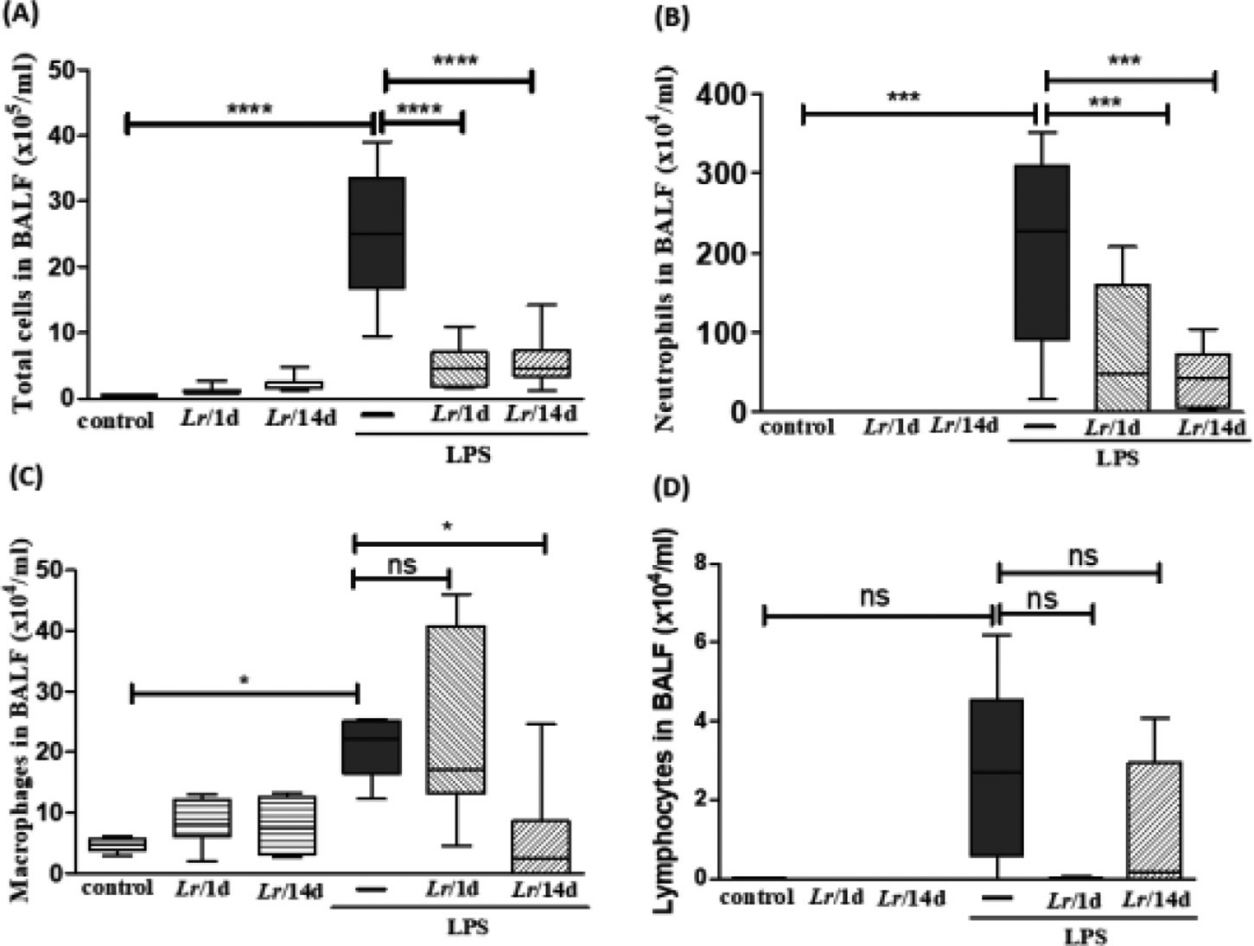
Total and differential of inflammatory cells in BALF. The C57Bl/6 mice were treated with *Lacticaseibacillus rhamnosus* (10^9^ CFU *Lr* in 300 µL of PBS, orogastric by gavage) for 14 days or one day, before the instillation of *E. coli* LPS (0.5 mg/Kg, intranasal). The mice were euthanized 24 h after the LPS, and the BALF was processed according to Materials and Methods. Total cells (2A); neutrophils (2B); Macrophages (2C); lymphocytes (2D). The results are expressed as mean ± SEM ****p < 0.0001; ***p < 0.001; **p < 0.01; *p < 0.05 and ns: non-significant difference.

Fig. 2 illustrates a significant increase in the total number of cells (2A), as well as neutrophils (2B) and macrophages (2C), in mice exposed to LPS. In the groups pre-treated with *Lr*, the total number of cells, neutrophils, and macrophages had a significant reduction. Regarding the lymphocyte population (2D), there was no change in any of the groups. The results did not indicate a significant difference between the groups' control, *Lr*/1d and *Lr*/14d.

### Neutrophils and lymphocytes in blood

**Fig. 3:**
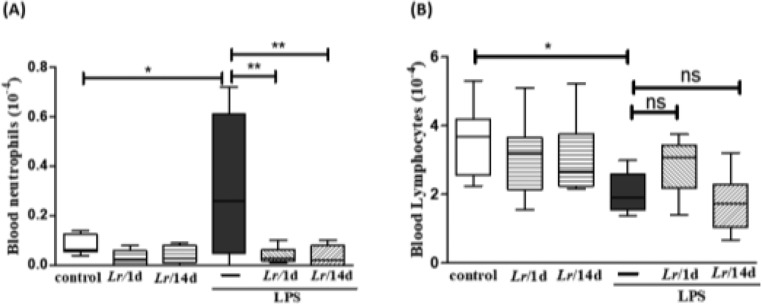
Leukocytes in blood. The C57Bl/6 mice were treated with *Lacticaseibacillus rhamnosus* (10^9^ CFU of *Lr* in 300 µL of PBS, gastric oro by gavage) for 14 days or one day, before the instillation of *E. coli* LPS (0.5 mg/Kg, intranasal). The mice were euthanized 24 h after the LPS and the whole blood analysis (white and red) was performed in according to Materials and Methods. Neutrophil in blood (3A) and Lymphocytes in blood (3B). The results are expressed as mean ± SEM ****p < 0.0001; ***p < 0.001; **p < 0.01; *p < 0.05 and ns, non-significant difference.

Fig. 3 highlights the effect of *Lr* on the population of both neutrophils and lymphocytes in the blood of mice from control, LPS, *Lr*/1d+LPS, and *Lr*/14d+LPS groups. The LPS-exposed mice had a greater number of neutrophils (3A) in their blood than the control group, and conversely, the *Lr* attenuated the neutrophils population in blood compared to the LPS group. The treatment strategies with *Lr* did not change the number of blood lymphocytes (3B) compared to the LPS group. The results did not indicate a significant difference between the groups' control, *Lr*/1d and *Lr*/14d.

### Expression of TLR2 and TLR4 in lung tissue

**Fig. 4:**
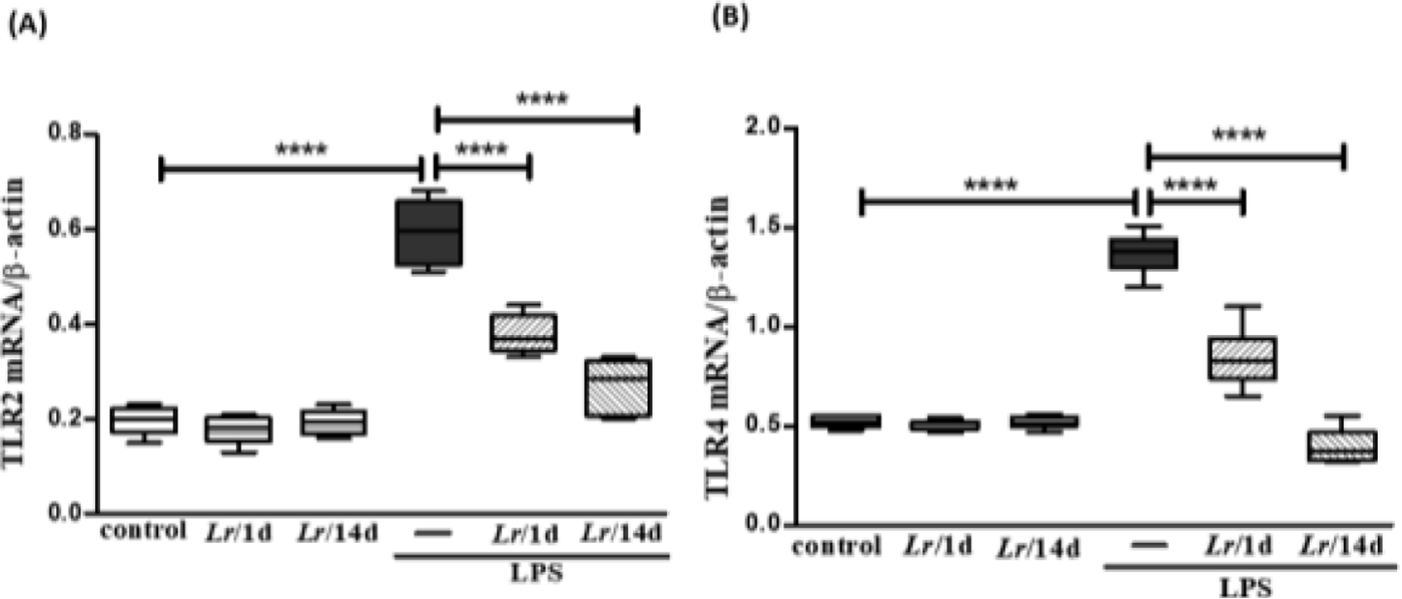
Gene expression of TLR2 and TLR4 receptors in lung tissue. The C57Bl/6 mice were treated with *Lacticaseibacillus rhamnosus* (10^9^ CFU of *Lr* in 300 µL of PBS, gastric oro by gavage) for 14 days or one day, before the instillation of *E. coli* LPS (0.5 mg/Kg, intranasal). The mice were euthanized 24 h after the LPS, and the qPCR was processed according to Materials and Methods. TLR2 (4A) and TLR4 (4B). The results are expressed as mean ± SEM ****p < 0.0001; ***p < 0.001; **p < 0.01; *p < 0.05 and ns: non-significant difference.

Fig. 4 illustrates that exposure to LPS increased the expression of mRNA of TLR2 (4A) and TLR4 (4B) in lung tissue when compared with the control group. On the other hand, *Lr* treatment reduced mRNA levels for both TLRs compared to mice exposed to LPS. The results did not indicate a significant difference between the groups' control, *Lr*/1d and *Lr*/14d.

### Cytokines in bronchoalveolar lavage fluid

**Fig. 5.**
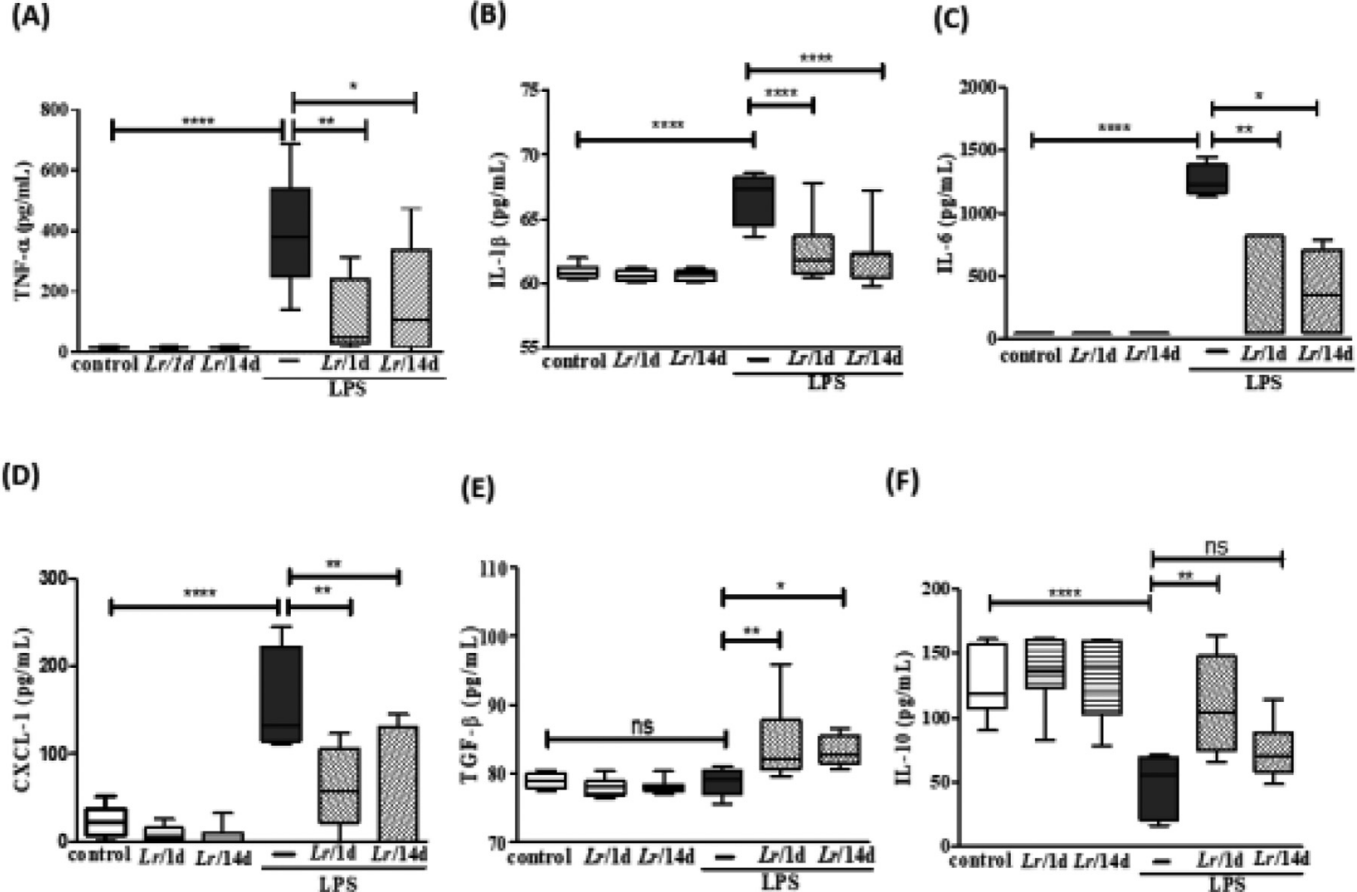
Cytokines in the BALF. The C57Bl/6 mice were treated with *Lacticaseibacillus rhamnosus* (10^9^ CFU *Lr* in 300 µL of PBS, orogastric by gavage) for 14 days or one day, before *E. coli* LPS instillation (0.5 mg/Kg, intranasal). The mice were euthanized 24 h after the LPS, and the BALF was processed according to Materials and Methods. TNF-α (5A); IL-1β (5B); IL-6 (5C); CXCL-1 (5D); TGF-β (5E), and IL-10 (5F). The results are expressed as mean ± SEM ****p < 0.0001; ***p < 0.001; **p < 0.01; *p < 0.05 and ns: non-significant difference.

Fig. 5 illustrates a marked increase in the concentration of TNF-α (5A), IL-1β (5B), IL-6 (5C), and CXCL-1 (5D) in BALF from LPS-exposed mice compared to the group control, and a significant decrease of these same cytokines when treated with *Lr*. Interestingly, the level of TGF-β (5E) showed no difference between the control and LPS groups. However, the same figure illustrates that *Lr* pre-treatment strategies increased the concentration of TGF-β in the BALF of mice exposed to LPS. The concentration of IL-10 (5F) was reduced in mice exposed to LPS compared to the control group. Furthermore, only in the *Lr*/1d+LPS group, the IL-10 level was restored to values close to those found in the control group. The results did not indicate a significant difference between the groups' control, *Lr*/1d and *Lr*/14d.

### Expression of metalloproteases and TIMP in lung tissue

**Fig. 6.**
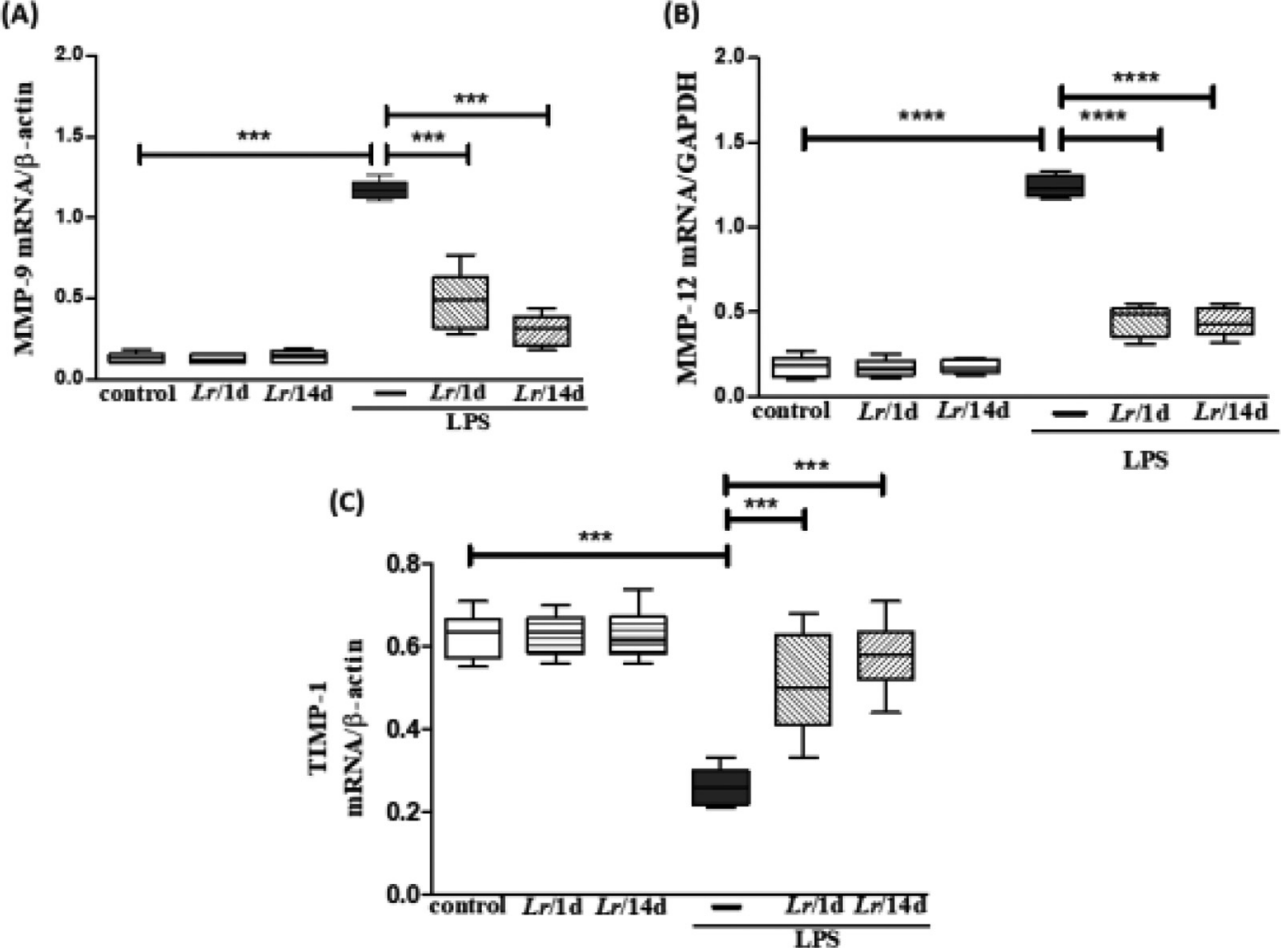
Gene expression of MMP-9, MMP-12 and TIMP in lung tissue. The C57Bl/6 mice were treated with *Lacticaseibacillus rhamnosus* (10^9^ CFU *Lr* in 300 µL of PBS, orogastric by gavage) for 14 days or one day, before *E. coli* LPS instillation (0.5 mg/Kg, intranasal). The mice were euthanized 24h after the LPS, and the qPCR was processed according to Materials and Methods. MMP-9 (6A); MMP-12 (6B), and TIMP (6C). The results are expressed as mean ± SEM ****p < 0.0001; ***p < 0.001; **p < 0.01; *p < 0.05 and ns: non-significant difference.

Fig. 6 represents the expression of metalloproteases MMP-9 (6A), MMP-12 (6B), and TIMP-1 (6C) shows the expression in LPS-exposed mice and pre-treated with *Lr* or not. Fig. 6A and 6B both illustrate that LPS significantly increased the expression of MMP-9 and MMP-12, respectively, compared to the control group. In contrast, *Lr* treatment strategies reduced mRNA levels for both metalloproteases in the lung tissue of mice from the LPS group. Otherwise, the TIMP-1 (6C) expression in lung tissue of the LPS group was markedly dropped compared to the control group, and the *Lr* restored TIMP-1 to levels close to the control group. The results did not indicate a significant difference between the groups *Lr*/1d+LPS and *Lr*/14d+LPS in the expression of MMP-12 (6B). The results did not indicate a significant difference between the groups' control, *Lr*/1d and *Lr*/14d.

### Lung oedema

**Fig. 7.**
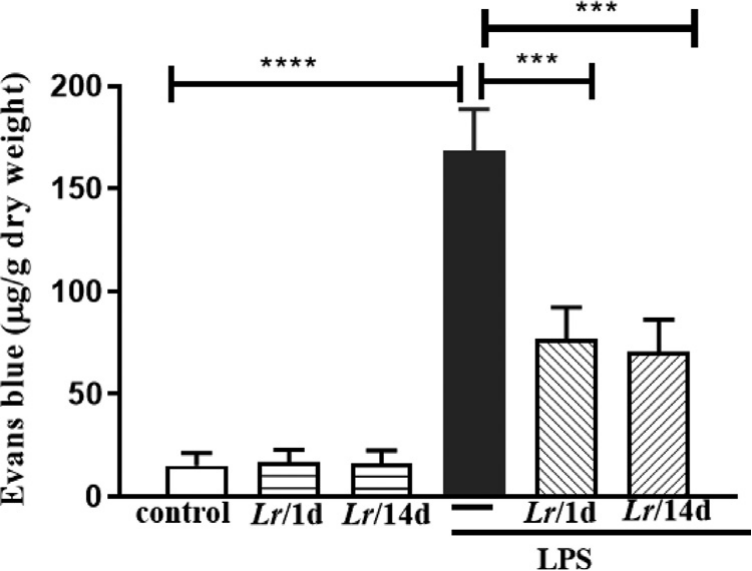
Lung edema. The C57Bl/6 mice were treated with *Lacticaseibacillus rhamnosus* (10^9^ CFU *Lr* in 300 µL of PBS, orogastric by gavage) for 14 days or one day, before *E. coli* LPS instillation (0.5 mg/Kg, intranasal). The mice were euthanized 24 h after the LPS. Pulmonary vascular permeability was assessed by Evans blue dye extravasation, processed according to Materials and Methods. The results are expressed as mean ± SEM ****p < 0.0001; ***p < 0.001; **p < 0.01; *p < 0.05 and ns: non-significant difference.

Fig. 7 represents the pulmonary edema in response to LPS instillation. In this assay, the authors determined vascular permeability in the lung using the Evans blue extravasation technique 24 h after LPS. The Evans blue extravasation into the lung was significantly higher in comparison with the control group. It is observed that both *Lr*/1d+LPS and *Lr*/14d+LPS significantly reduced pulmonary microvascular leakage 24 h after LPS. The results did not indicate a significant difference between the groups' control, *Lr*/1d and *Lr*/14d.

### Morphometry in lung

**Fig. 8.**
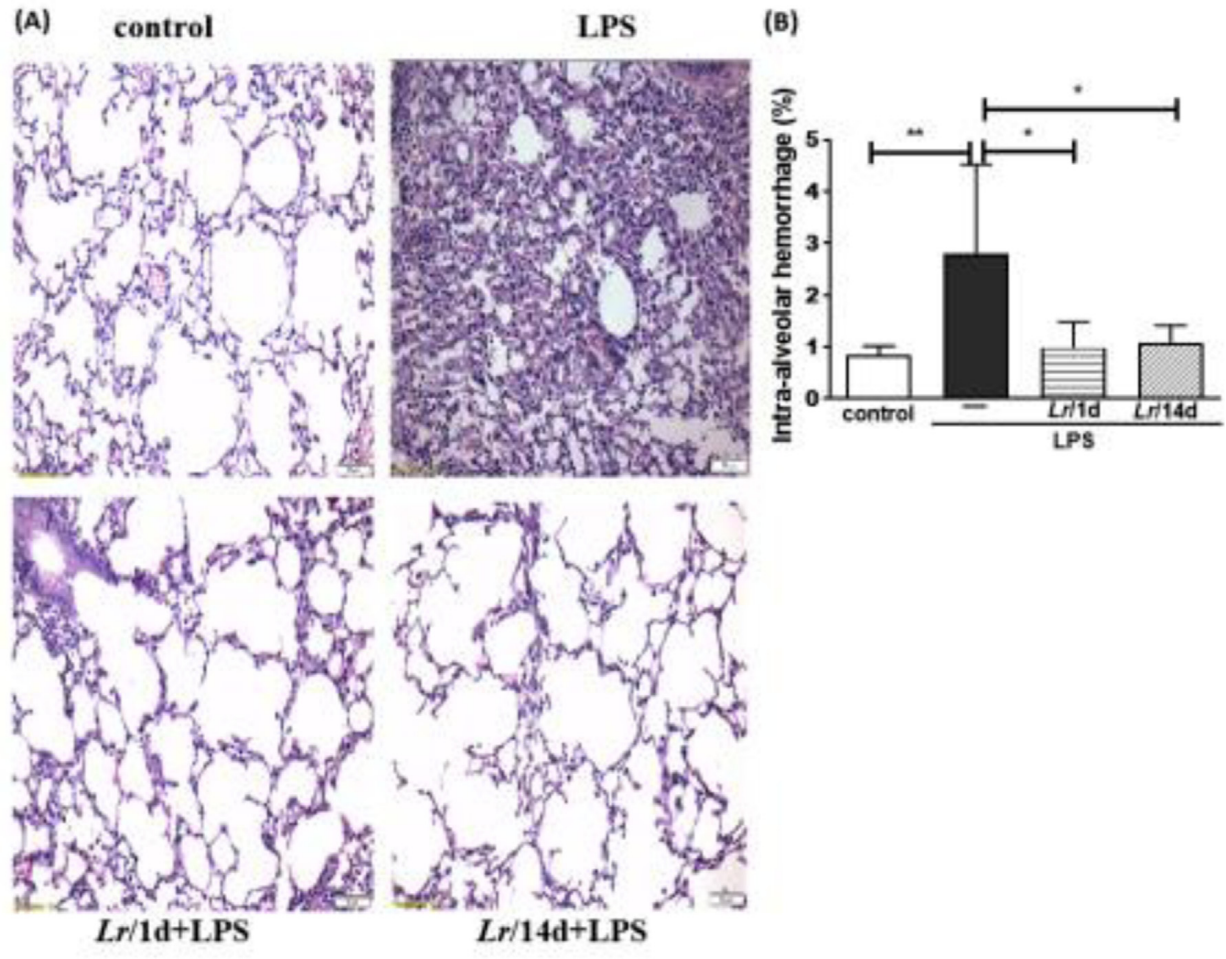
Lung morphometry (200 ×). The C57Bl/6 mice were treated with *Lacticaseibacillus rhamnosus* (10^9^ CFU *Lr* in 300 µL of PBS, orogastric by gavage) for 14 days or one day, before *E. coli* LPS instillation (0.5 mg/Kg, intranasal). The mice were euthanized 24 h after the LPS. The histological sections were stained with H&E and the photomicroscopy of the parenchyma was performed using a microscope (OLYMPUS MODEL BX43F) and analyzed using the IMAGE PRO PLUS program. Photomicroscopy (magnification: 200 ×) (8A); haemorrhage (8B). The results are expressed as mean ± SEM ****p< 0.0001; ***p< 0.001; **p< 0.01; *p< 0.05 and ns: non-significant difference.

**Fig. 9.**
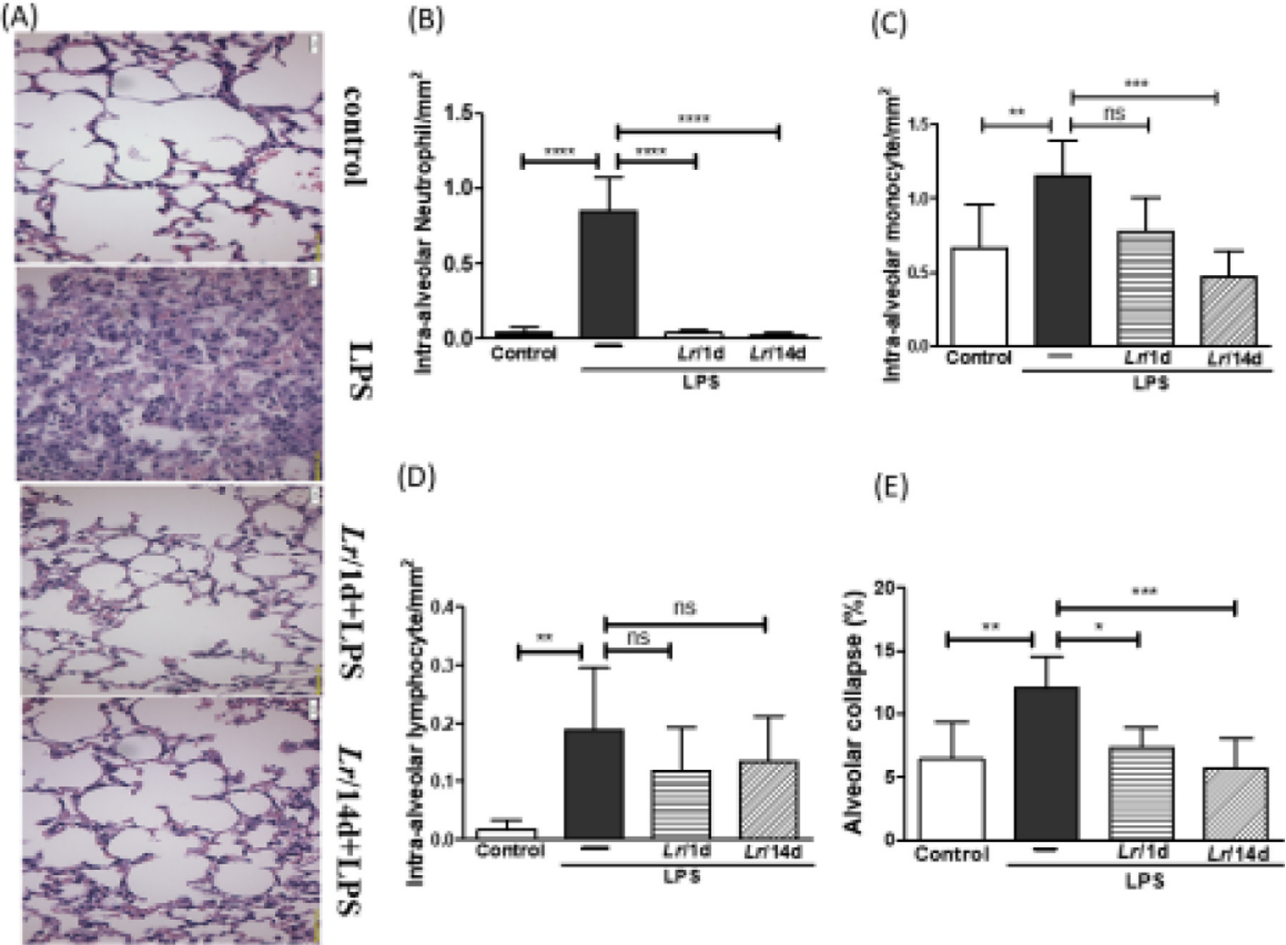
Morphometry in lung (400 ×). The C57Bl/6 mice were treated with *Lacticaseibacillus rhamnosus* (10^9^ CFU *Lr* in 300 µL of PBS, gastric oro by gavage) for 14 days or one day, before *E. coli* LPS instillation (0.5 mg/Kg, intranasal). The mice were euthanized 24 h after the LPS. The histological sections were stained with H&E and the photomicroscopy of the parenchyma was performed using a microscope (OLYMPUS MODEL BX43F) and analyzed using the IMAGE PRO PLUS program. Photomicroscopy (magnification: 400 ×) (9A); Neutrophils (9B); monocytes (9C); lymphocytes (9D), and Alveolar collapse (9E). The results are expressed as mean ± SEM ****p < 0.0001; ***p < 0.001; **p < 0.01; *p < 0.05 and ns: non-significant difference.

Fig. 8A illustrates the photomicroscopy of each experimental group: control, LPS, *Lr*/1d+LPS, and *Lr*/14d+LPS. Fig. 8 shows the alveolar hemorrhage (8B), were elevated in mice exposed to LPS compared to the control group, data represented in photomicroscopy (200 ×). In addition, photomicroscopy (400 ×) represented in Fig. 9A made it possible to measure the increase in populations of neutrophils (9B), monocytes (9C), lymphocytes (9D), as well as the alveolar collapse (9E) in the lung tissue of mice in the LPS group compared to the control group. In contrast, *Lr* pre-treatment reduced all changes in LPS-induced lung morphometry, except for the number of lymphocytes in the intra-alveolar space. In addition, *Lr* was more efficient when administered for 14 days compared to animals that received the probiotic one day before LPS.

### Bronchial hyperreactivity

**Fig. 10.**
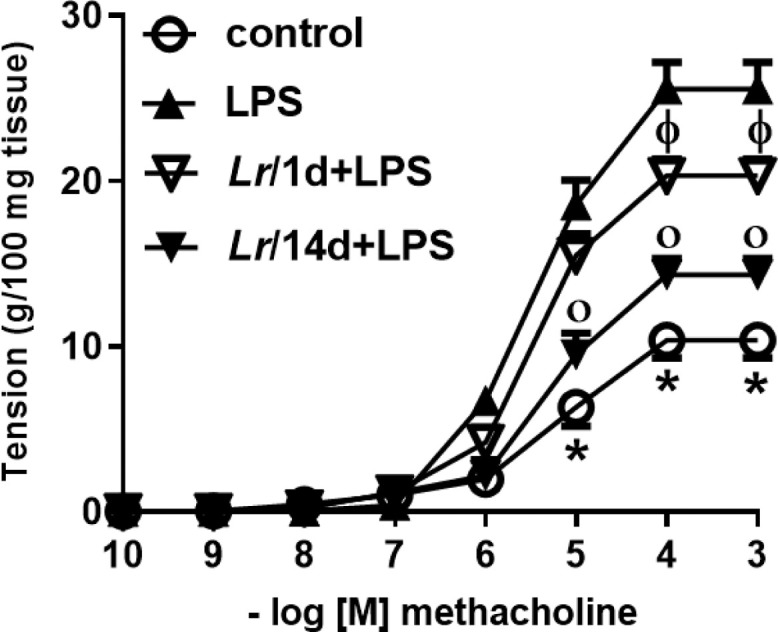
Bronchial hyperreactivity. The C57Bl/6 mice were treated with *Lacticaseibacillus rhamnosus* (10^9^ CFU of *Lr* in 300 µL of PBS, orogastric by gavage) for 14 days or one day, before *E. coli* LPS instillation (0.5 mg/Kg, intranasal). The mice were euthanized 24 h after the LPS. The bronchial hyperreactivity were processed according to Materials and Methods. Measurement of BSM contraction forces were performed by varying the concentration of MCh-levels from 10‒10 to 10‒3M. The results are expressed as mean ± SEM. Statistically significant differences *p* < 0.05 comparing the groups (control, *Lr*/1d+LPS, *Lr/*14d+LPS) with the LPS group are represented by the symbols in the figure.

Fig. 10 represents the maximal contractile force of bronchial segments from mice from control, LPS, *Lr/*1d+LPS, and *Lr*/14d+LPS. The bronchial segments were stimulated with increasing doses (10^−10^ to 10^−3^M) of methacholine, a cholinergic agonist, and then a concentration-response curve was performed. Fig. 10 illustrates that elapsed 24 h of LPS instillation, the maximal contractile response of bronchi segments to methacholine increased compared to the control group. On the contrary, previous treatment with *Lr*1 day before LPS (*Lr*/1d+LPS), as well as Lr 14 days before LPS (Lr/14d+LPS), attenuated maximal contractile response against methacholine compared to the LPS group.

### NF-κB in lung tissue

**Fig. 11.**
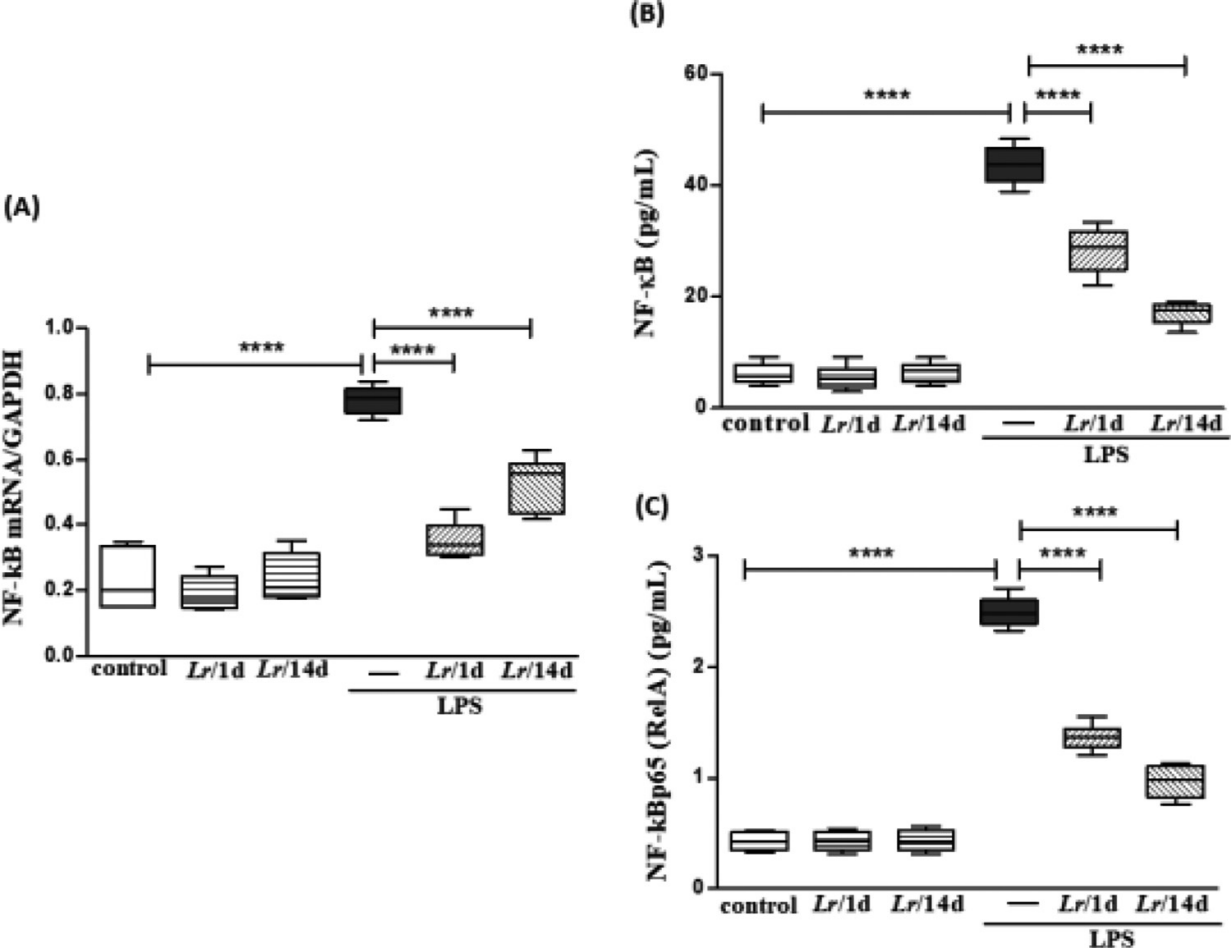
Gene expression of NF-kB and protein concentration of NF-κB and NF-κB p65 (RelA) in lung tissue. The C57Bl/6 mice were treated with *Lacticaseibacillus rhamnosus* (10^9^ CFU of *Lr* in 300 µL of PBS, orogastric by gavage) for 14 days or one day, before *E. coli* LPS instillation (0.5 mg/Kg, intranasal). The mice were euthanized 24 h after the LPS, and the homogenate and qPCR were processed according to Materials and Methods. mRNA (11A); NF-kB (11B), and NF-kBp65 (RelA) (11C). The results are expressed as mean ± SEM ****p < 0.0001; ***p < 0.001; **p < 0.01; *p < 0.05 and ns, non-significant difference.

Fig. 11 illustrates that exposure to LPS increased the expression of mRNA (11A) as well as the protein concentration of NF-κB (11B) and NF-κB p65 (RelA) (11C) in lung tissue compared with the control group. Conversely, *Lr* pre-treatment strategies reduced gene expression and protein concentration of NF-kB/NF-kBp65 when compared to the LPS group. The results did not indicate a significant difference between the groups' control, *Lr*/1d and *Lr*/14d.

## Discussion and conclusion

*Lacticaseibacillus rhamnosus* is a non-pathogenic bacterium commonly used to relieve some gastrointestinal disturbs due to its ability to maintain the equilibrium of intestinal flora.^29^ Previous studies have shown a beneficial effect of *Lr* in attenuating inflammatory conditions due to colitis,^30^ allergic asthma,^31^ and pneumonia.^32^ Additionally, some authors showed that endotoxemic mice[33] and patients with sepsis-induced pneumonia presented a marked reduction of airway inflammation associated with improved lung function.^19^ That is one of the reasons why the *Lactobacillus* are gaining prominence in the treatment of chronic inflammatory diseases, such as asthma[17] and COPD.^34^ To extend the previous findings, the present manuscript investigated for the first time two strategies of probiotic therapy in a murine model of ALI induced by lipopolysaccharide from *Eschericia coli*.

The present study shows that a daily oral diet with *Lacticaseibacillus rhamnosus* for a short time modulates the inflammatory response in an *in vivo* model of ALI. Some authors describe that these non-pathogenic bacteria modulate both the inflammatory and immune responses in distant organs and thus could be more efficient than other pharmacological therapies.^31^ Probiotics are considered a nonspecific adjuvant of the innate immune response, helping the body's defenses promote a suitable immune response, depending on pathological condition, and restore the balance between pro-and anti-inflammatory chemokines secreted by activated immune cells. Thus, most studies reveal that probiotics are devoid of side-effects compared to conventional pharmacological therapy for ALI treatment.^1^

The present manuscript indicates that the probiotic beneficial effect is triggered quickly even when lung inflammation is an acute response. The present study's data evidence a protective effect of *Lr*, 1 and 14 days before LPS exposure in attenuating both the lung inflammation and the neutrophils population in blood. The present study's findings corroborate previous studies showing that LPS induces a robust migration of inflammatory cells to the lung environment, mainly macrophages and neutrophils.^4^ In response to LPS, pulmonary cells produced pro-inflammatory cytokines, such as IL-1β, IL-6, and TNF-α, resulting in the secretion of several chemokines. The increase in CXCL1 levels promotes the migration and differentiation of monocytes in lung tissue, amplifying the inflammatory process.^35^ Otherwise, the pro-inflammatory cytokines CXCL1, TNF-α, IL-1β, and IL-6 secreted into BALF from LPS-challenged mice were markedly reduced after both treatment strategies with *Lr*.

For TLRs, the present study's findings demonstrate that some sensors of innate immunity, such as TLR2 and TLR4, are involved in the beneficial effect of both therapeutic strategies with probiotics. This is an important finding because the TLRs are responsible for recognizing the LPS to initiate the triggering of cell signaling that involves the phosphorylation of NF-κB and hence the secretion of pro-inflammatory proteins in the lung environment.^36^ The authors’ results found a better performance of *Lr* on the inflammatory infiltrated in BALF as well as the TLR2 and TLR4 expression in lung tissue when the LPS-exposed mice were treated 14 days before LPS.

The acute inflammation of the lungs also results in alterations in the parenchyma architecture, a process known as tissue remodeling, due to an unbalance between active MMPs and their inhibitors, TIMP.^8^^,^^37^ In concordance with this notion, the LPS group presented, in association with pulmonary neutrophilia, intra-alveolar hemorrhage, and alveolar collapse. These structural alterations were accomplished by a significant increase in the mRNA expression for MMP-9 and MMP-12 that was inverse to gene expression for TIMP. Despite the cytokine/chemokine storm and the consequent cascade of events induced by LPS, probiotic feeding attenuated the inflammatory process both in the lung edema intra-alveolar hemorrhage. The effect of the *Lr* pre-treatment only one day before LPS on MMP/TIMP balance in the LPS group indicates that the *Lr* can reprogram the lung milieu, just 24 h before LPS, to face the changes in lung morphometry, which may compromise the respiratory mechanics.

It is important to highlight that in ALI/ARDS the increase in capillary permeability and extravascular fluid accumulation protein-rich edema, arising from an inflammatory response, is an important pathophysiological mechanism and frequently is associated with the presence of neutrophils and their products, which play an important pathogenic role in ARDS.^23^ Pulmonary edema from increased alveolar-capillary barrier permeability compromises gas exchange and lung mechanical properties, including decreased total lung compliance, followed by bronchoconstriction and hyperresponsiveness in response to methacholine.^38^ These findings corroborate with the present study's results since the LPS group presented vascular permeability higher than the control group. On the contrary, previous treatment with *Lr* markedly decreased pulmonary edema in mice exposed to LPS. These results suggest that *Lr* can interact with cell mechanisms that orchestrate changes in the architecture of pulmonary endothelial cytoskeleton in sepsis insult.

Although septic patients do not present bronchoconstriction crisis, such as in asthma, there is an increase of contractile tonus in bronchial segments of sepsis individuals.^39^ It is due to some pro-inflammatory mediators, such as TNF-α and IL-1β. These cytokines are produced by a variety of cells in the airways, and diverse authors evidenced a marked involvement of TNF-α and IL-1β with airway inflammation and bronchial hyperresponsiveness when stimulated with cholinergic agonists.^38^^,^^40^ The present study's results corroborate with these findings because levels of both TNF-α and IL-1β are elevated in BALF of LPS group. In contrast, pre-treated mice with *Lr* had a decrease of TNF-α and IL-1β levels in BALF as well as bronchial hyperreactivity to methacholine compared to their respective LPS groups. These data about bronchial hyperreactivity can be interpreted as direct interaction of probiotic metabolites with bronchial smooth muscle cells or as an indirect effect due to significant reduction of both cellularity and secretion of pro-inflammatory mediators in BALF after pre-treatment with *Lr*. In fact, further studies are needed to assess the mechanism by which PBM attenuates bronchial hyperreactivity.

Although the mechanisms behind this effect are allusive, the authors found that *Lr* feeding restored both the levels of IL-10 and TGF-β in the BALF to the level of control animals, indicating that probiotics induced an anti-inflammatory stead state. Although TGF-β is known as a potent pro-fibrotic factor, some authors have demonstrated that this cytokine presents pleiotropic effects.^41^ In fact, the TGF-β has been shown as a cytoskeleton integrity protector of airways because it is involved with the interaction of gap-junctions between these lung cells.^42^ Following that thought, the authors’ results evidenced that both therapeutic strategies with *Lr* increased the TGF-β secretion in BALF of mice from the LPS group, which could be a reflex effect of *Lr* that attenuates both the alveolar hemorrhage and the intra-alveolar neutrophilic infiltrated. The present study's results show a discrete reduction of TGF-β levels in the LPS group but without statistical difference because other inflammatory mediators can induce lung edema and hemorrhage in sepsis.^43^

Otherwise, the IL-10 levels in BALF of mice from the LPS group presented a marked fall. This result is in accordance with authors, who evidence that the series of acute inflammatory events in lung inflammation induced by LPS or sepsis do not allow the secretion of IL-10, a regulator protein recognized by its anti-inflammatory properties, accompanying the storm of pro-inflammatory cytokines in lung environmental.^44^ Among therapeutic strategies with *Lr* on IL-10 secretion in the LPS group studied here, the probiotic administered only one day before LPS restored the IL-10 levels, indicating that the anti-inflammatory stead-state of IL-10 induced by probiotic pre-treatment seems to be acute. Otherwise, the pre-treatment effect with *Lr* on pro-inflammatory cytokines was efficient in both strategies. Although the pre-treatment with *Lr*14 days before LPS did not restore IL-10 secretion, the increase of this anti-inflammatory cytokine occurred while all evaluated pro-inflammatory cytokines were also upregulated by LPS. However, a therapeutic limitation of pre-treatment with *Lr* should be considered, seeing that the probiotic regulatory effect was not maintained by only treatment 14 days before LPS.

The purpose of this study study is not to establish the *Lr* probiotic as a specific therapy in the treatment of sepsis. Probiotics are non-pathogenic bacteria that have the ability to modulate the immune response of organs distant from the intestine, such as the lung. In addition, some studies show that supplementing the diet with a probiotic can help robustness the immunological system, and it gives probiotics the ability to create protection in lung microenvironmental against inflammatory response still not started.^32^ Thus, the purpose of the study is that healthy individuals use probiotics in daily routine, and thus it would control the inflammatory response when it is exacerbated, such as in a sepsis condition. Regarding the effects of probiotics after ALI induced by LPS, there are some authors show that probiotics attenuate the incidence of VAP ventilator-associated pneumonia, as well as the expansion of pathogenic intestinal bacteria.^45^ Moreover, some authors evidenced priming the immune system for a robust immune response and increased production of beneficial microbial products such as short-chain fatty acids.^15^ In addition, probiotics can enhance immune activity, decrease the occurrence of nosocomial pneumonia and multiple organ dysfunction syndromes, and reduce days in the hospital.^46^ These results corroborate findings that show a decrease in sepsis incidence, to improve sepsis outcome, and to decrease late mortality after sepsis.^33^ Despite the beneficial effect of probiotics after ALI, it is important to highlight that regimen of previous treatment creates an anti-inflammatory lung milieu, and this condition can alleviate inflammatory response in the presence of sepsis. It is important to consider that sepsis is a systemic inflammatory response in which the severity of symptoms is acute and culminates in multiple organ failure. For this reason, therapies capable of modulating the immune system of healthy individuals enable an adequate inflammatory response in the presence of sepsis.

Although animal studies have provided clear evidence that lactobacillus can have profound immunoregulatory effects and regulate immune responses beyond the gastrointestinal tract, the results of clinical trials have been highly variable. Therefore, it is important to consider which strains of bacteria can produce the most efficient immune regulation in accordance with the immunologic disorder. Further studies are needed to investigate whether a single strain of bacteria can control disease-specific characteristics or if probiotics are homeostasis regulators capable of inducing a regulatory immune response according to the disease milieu.

Taken together, the authors’ studies indicate that the oral feeding with *Lr* modulates in a short pre-treatment period exerts a response in the host immune by preventing the lung inflammation, associated principally with the lung neutrophilia and alveolar hemorrhage and edema, via downregulation of NF-κB transcription factor. The *Lr* also restores the regulatory response via the upregulation of IL-10 secretion. Finally, these data indicate that the probiotic *Lacticaseibacillus rhamnosus* could be promising for the development of new strategies to partially prevent ALI from sepsis.

## CRediT authorship contribution statement

**Fabiana Olimpio:** Conceptualization, Investigation, Project administration, Writing – review & editing. **José Roberto Mateus da Silva:** Project administration. **Rodolfo P. Vieira:** Project administration. **Carlos R. Oliveira:** Project administration. **Flavio Aimbire:** Data curation, Methodology, Writing – original draft, Writing – review & editing.

## Conflicts of interest

The authors declare no conflicts of interest.
